# Succinylation-associated lncRNA signature to predict the prognosis of colon cancer based on integrative bioinformatics analysis

**DOI:** 10.1038/s41598-023-34503-2

**Published:** 2023-05-05

**Authors:** Si-ming Zhang, Cheng Shen, Jue Gu, Jing Li, Xiaohui Jiang, Zhijun Wu, Aiguo Shen

**Affiliations:** 1 Cancer Research Center, Affiliated Tumor Hospital of Nantong University, Nantong, Jiangsu Province China; 2 Department of Computer Science and Engineering, Tandon School of Engineering, New York University, Brooklyn, USA; 3 Affiliated Hospital of Nantong University, Nantong, China; 4 Department of General Surgery, Affiliated Tumor Hospital of Nantong University, Nantong, China; 5 Department of Oncology, Nantong Second People’s Hospital, Nantong, China

**Keywords:** Cancer, Computational biology and bioinformatics, Biomarkers, Gastroenterology, Risk factors

## Abstract

Colon cancer (CC) has a poor 5-year survival rate though the treatment techniques and strategies have been improved. Succinylation and long noncoding RNAs (lncRNAs) have prognostic value for CC patients. We analyzed and obtained succinylation-related lncRNA by co-expression in CC. A novel succinylation-related lncRNA model was developed by univariate and Least absolute shrinkage and selection operator (Lasso) regression analysis and we used principal component analysis (PCA), functional enrichment annotation, tumor immune environment, drug sensitivity and nomogram to verify the model, respectively. Six succinylation-related lncRNAs in our model were finally confirmed to distinguish the survival status of CC and showed statistically significant differences in training set, testing set, and entire set. The prognosis of with this model was associated with age, gender, M0 stage, N2 stage, T3 + T4 stage and Stage III + IV. The high-risk group showed a higher mutation rate than the low-risk group. We constructed a model to predict overall survival for 1-, 3-, and 5-year with AUCs of 0.694, 0.729, and 0.802, respectively. The high-risk group was sensitive to Cisplatin and Temozolomide compounds. Our study provided novel insights into the value of the succinylation-related lncRNA signature as a predictor of prognosis, which had high clinical application value in the future.

## Introduction

With the development of society and change of lifestyle, the incidence of colon cancer (CC) is gradually increasing^1,2^. CC is one of the most common malignancies that accounts for the second highest number of new cancer cases in women and the third highest number of cancer diagnoses in men worldwide^3^. Therefore, it is particularly important to identify the potential mechanisms of action that promote the progression of CC and to discover new biomarkers.

Posttranslational modifications (PTMs) such as classical phosphorylation, ubiquitination and recently discovered acetylation, succinylation, SUMOylation, butyrylation, lactylation, etc.^4–6^ were known to play various important roles in the formation and development of different types of tumors. Lysine succinylation is a process during which succinyl groups are transferred from succinyl-CoA to specific alpha-amino residues. Recently, CPT1A promoted the proliferation of breast cancer^7^ gastric cancer^8^ through enolase 1 succinylation and succinylation of S100A, respectively. In pancreatic ductal adenocarcinoma, KAT2A promotes proliferation and migration by upregulating 14-3-3ζ via KAT2A succinyltransferase activity^9^. However, succinylation studies mainly focused on the basic filed, most of which only regulated underlying mechanism of a specific succinylase on the substrate protein in the CC. Long non-coding RNAs (lncRNAs) are the transcripts that have more than 200 nucleotides in length^10^, and play an important role in many biological functions, such as regulating post-translational modification. For example, lncPRESS1 is a p53-regulated lncRNA that silences the SIRT6 and mediates deacetylation of histone H3K56 and H3K9^11^. Moreover, the lncRNA binding to the NF-KB directly lead to the inhibition of phosphorylation of IKBB and NF-KB activation in breast cancer^12^. A variety of machine learning uses large databases to mine effective prognostic targets for predicting the prognosis of patients^13–15^, including CC^16^. However, the relationship of succinylation related lncRNAs and CC remained uncertain. Therefore, our study aimed to demonstrate the value of succinylation-related lncRNAs in CC patients.

## Materials and methods

### Data acquirement and identification of succinylation-related prognostic lncRNAs

**Figure 1:**
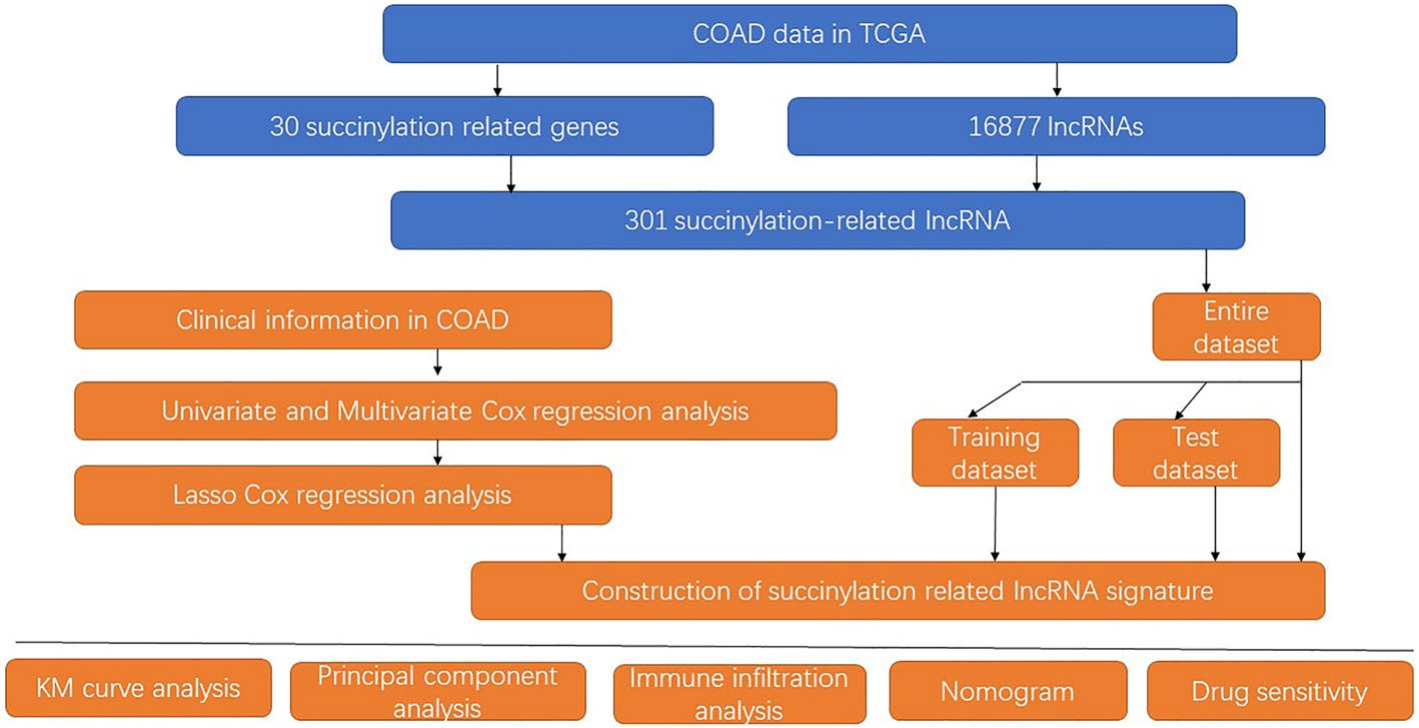
This study flow chart.

The analysis process of our study is shown in in Fig. 1. The transcriptome data of 473 patients with CC and 41 normal samples were downloaded from The Cancer Genome Atlas. The succinylation genes were obtained from GENCARDS (https://www.genecards.org/), we selected the top 30 genes including OXCT1, SUCLA2, SUCLG2, SUCLG1, OGDH, OXCT2, DLST, ACOT4, DLD, KAT2A, OGDHL, MMUT, SIRT5, ALAS2, SUGCT, ALAS1, DHTKD1, YEATS4, ACOT8, GAPDH, RYR1, MHS6, ACLY, NUDT7, NUDT8, NUDT19, ALB, HADHB and ACAA2. We identified lncRNAs that significantly related to the succinylation genes by Pearson’s correlation analysis in the R package ggalluvial.

### Establishing and validating a prognostic risk model

The entire set was randomly divided into two groups (training sets and test sets). We constructed a succinylation-related lncRNA model using the training set, test set and the entire set. The lncRNA signature to predict the prognosis of CC patients was established using Lasso analysis in the R package glmnet. The risk score of succinylation associated with lncRNA was calculated using the following formula: Risk Score = 0.66736 * FOXD3-AS1−0.84775 * ANK3-DT + 0.56176 * EIF3J-DT + 0.52115 * MIR210HG + 0.71575 * MAFA-AS1 + 1.24169 * AC024581.1 and based on the median risk score, low- and high-risk groups were divided.

### PCA and functional analysis

Principal component analysis is a statistical method of dimension reduction and grouping visualization of the entire profiles. We screened differentially expressed genes (DEGs) using GO analysis. The R package clusterProfiler was used in the analysis.

### Survival analysis with succinylation-related lncRNAs and evaluating model clinicopathology features

Succinylation-associated lncRNAs were subjected to perform survival analysis in the low- and high- risk groups using R software with survival package^17^, and survival related clinicopathology features were age, gender, TMN stage.

### Construction and evaluation of the nomogram

We established a nomogram between the risk score and clinicopathology features to predict 1-, 3-, and 5- year OS. Dependent on the Hosmer–Lemeshow test, we calculated modified curve to illustrate the consistency of the actual and predicted outcome. AUC and ROC curves were used to assess the clinicopathological features for prognosis.

### Exploration of potential chemotherapeutic drugs

To explore the succinylation-related lncRNAs for CC patients, we screened IC50 using package of R software and chemotherapy drugs from GDSC (Genomics of Drug Sensitivity in Cancer) database^18^.

## Results

### Identification of succinylation-related lncRNAs in colon tissue samples

**Figure 2:**
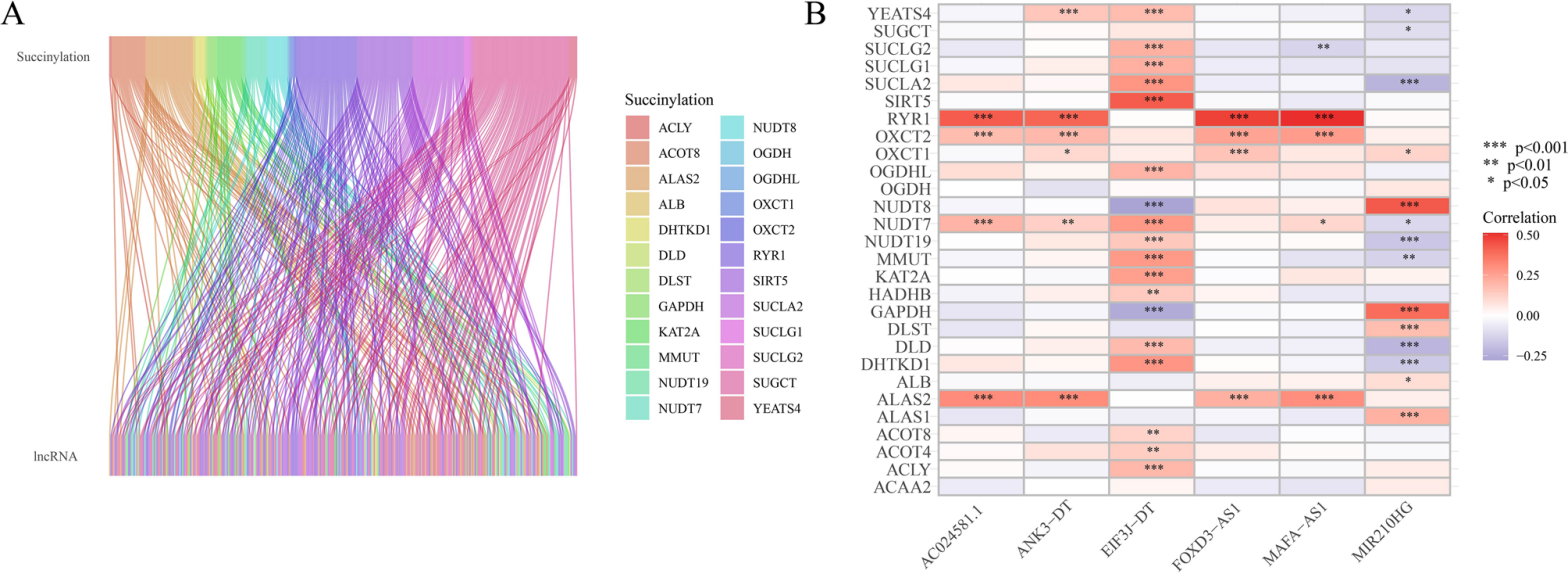
Correlation with succinylation-related lncRNAs. (**A**) Sankey plot of succinylation genes and succinylation-related lncRNAs. (**B**) the association with succinylation genes and succinylation-related lncRNAs expression in the heatmap (ns p > 0.05, *p < 0.05, **p < 0.01 and ***p < 0.001).

The matrix expression of top 30 succinylation genes and 16,877 lncRNAs was screened and then we selected succinylation-related lncRNAs by |Pearson R|> 0.4 and p < 0.001. Subsequently, we constructed a network of succinylation-lncRNA coexpression in the Sankey diagram (Fig. 2A). The correlation with succinylation genes and 300 succinylation-related lncRNA were shown in Fig. 2B.

### Construction a risk model using succinylation-associated lncRNAs for patients of CC

**Figure 3:**
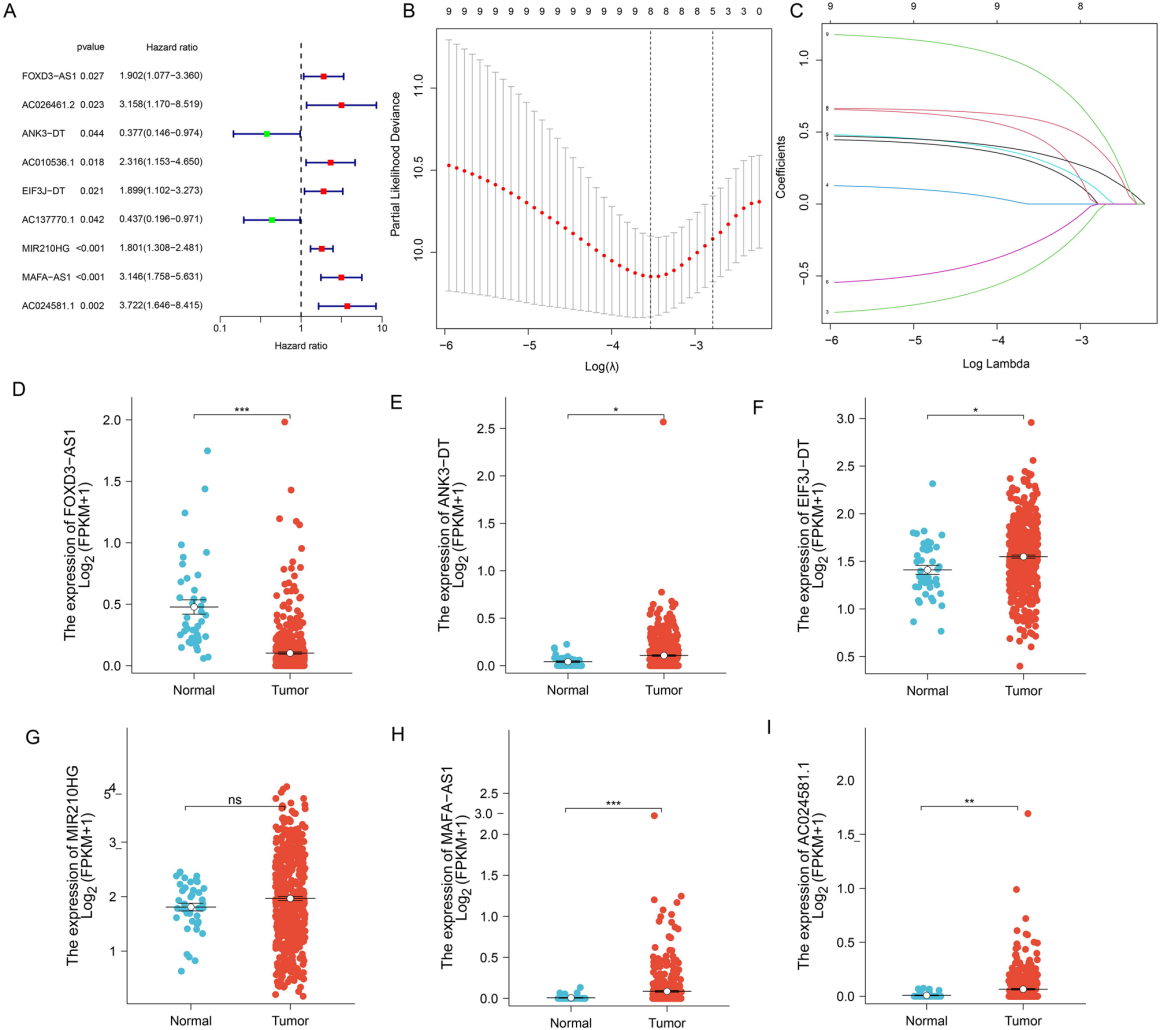
The risk model in colon patients. (**A**) Univariate Cox regression analysis of the succinylation-related lncRNAs. (**B** and **C**) Construction of the risk signature by select genes based on Lasso-Cox regression analysis. (**D**–**I**) The expression of these lncRNAs between normal tissues and tumor tissues. (ns p > 0.05, *p < 0.05, **p < 0.01 and ***p < 0.001).

We screened succinylation-related prognostic lncRNAs by univariate Cox regression analysis, and nine succinylation-related lncRNAs were found to be significantly correlated with OS in Fig. 3A. Lasso Cox regression analysis was used to find novel prognostic target for predicting clinical results while avoiding the occurrence of collinearity of transcriptome data^19^. Lasso cox analysis in the Fig. 3B and C and ultimately identified the six lncRNAs including FOXD3-AS1, ANK3-DT, EIF3J-DT, MIR210HG, MAFA-AS1, AC024581.1.

We then analyzed the expression of succinylation-related lncRNAs in the CC. The lncRNAs ANK3-DT, EIF3J-DT, MAFA-AS1 and AC024581.1 showed high expression in tumor tissues (Fig. 3E, F, H and I). However, lncRNA FOXD3-AS1 displayed a low expression in tumor tissues (Fig. 3D). LncRNA MIR210HG showed no difference in the CC patients (Fig. 3G).

**Figure 4:**
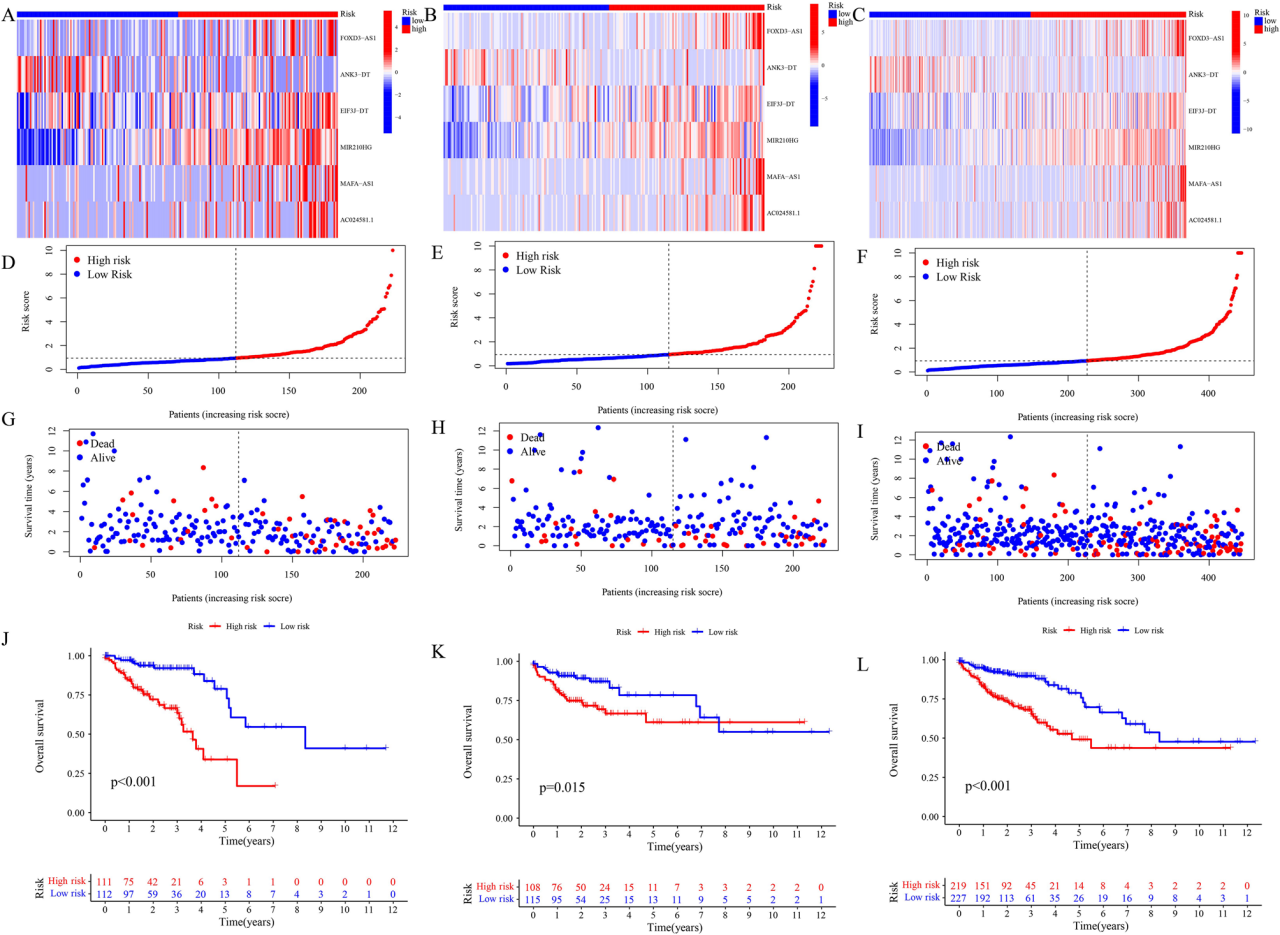
The risk patterns of prognostic value in six succinylation-related lncRNAs. (**A**–**C**) Training set, test set, and entire set analysis showing the expression of six prognostic lncRNA for each patient. (**D**–**F**) Distribution of succinylation-related lncRNAs for the training set, test set, and entire set. (**G**–**I**) Differences patterns in survival status and survival time of the training set, test set, and entire set. (**J**–**L**) K–M survival analysis by K–M curve for OS of patients.

To further examine whether the succinylation-related lncRNAs were related to prognostic capability, we assessed the risk scores in the training set, test set and entire set. Based on the median value of the three sets, six succinylation-related expressed lncRNAs in the high- and low-risk groups were shown in the heatmap (Fig. 4A–C). Patients were assigned into two group, with the risk score increased, the death rate rose and survival time decreased in the training set (Fig. 4D and G), test set (Fig. 4E and H) and entire set (Fig. 4F and I). CC patients in the high group r had a poor OS than those with the low group, as shown by the Kaplan–Meier survival analysis (training set: p < 0.001 shown in Fig. 4J; test set: p < 0.05 shown in Fig. 4K; entire set : p < 0.001 shown in Fig. 4L).

**Figure 5:**
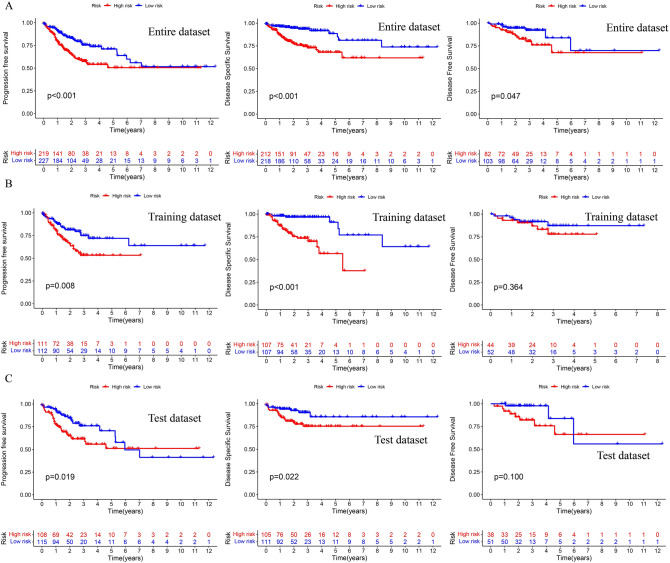
The risk signature of PFS, DSS, DFS in six succinylation-related lncRNAs in entire dataset (**A**), training dataset (**B**) and test dataset (**C**).

Subsequently, we explored the progression-free survival (PFS), disease-specific survival (DSS) and disease-free survival (DFS) between the high and the low groups in CC patients, respectively in entire dataset, training dataset, and test dataset (Fig. 5A–C), and found high-risk succinylation-related lncRNAs were related to a poor prognosis. These results indicated that succinylation-related lncRNAs signature had a great predictive value.

**Figure 6:**
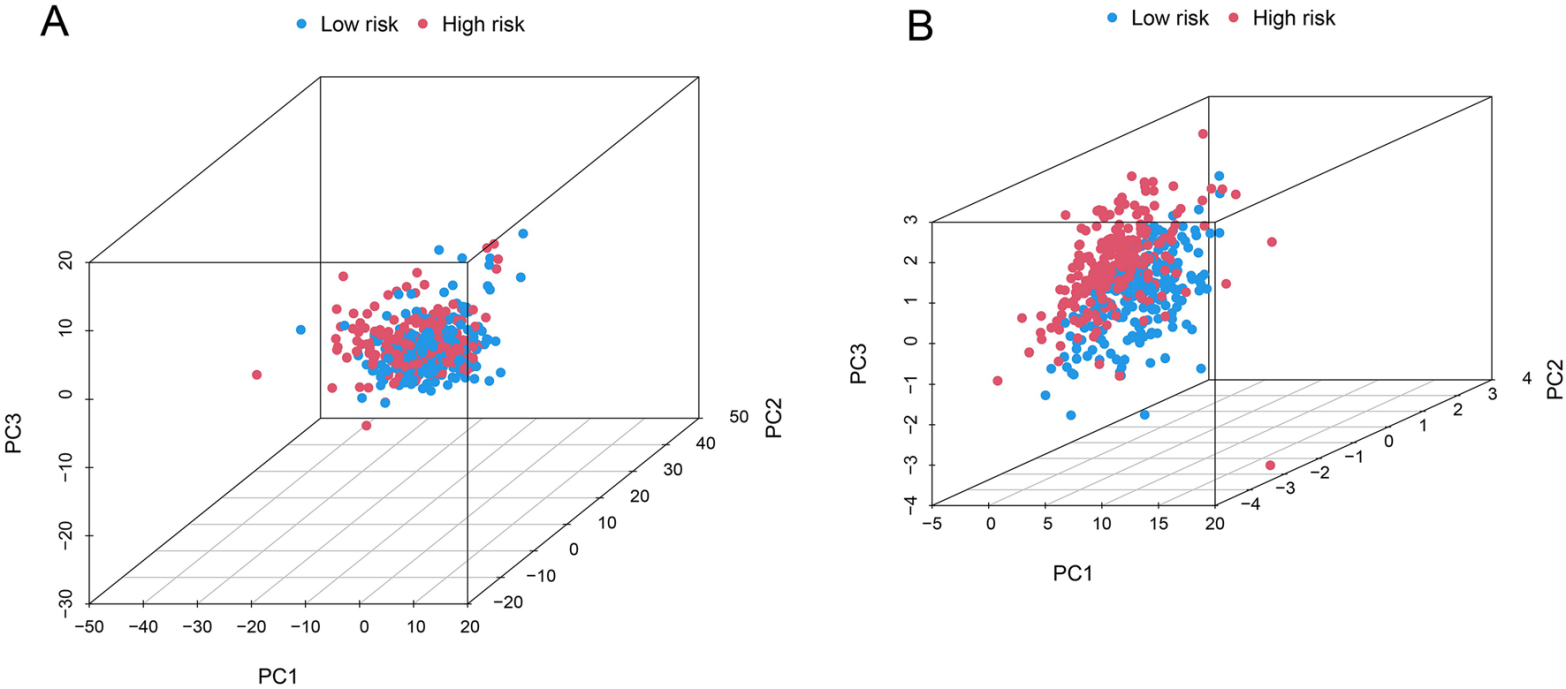
Principal component analysis (**A**) succinylation-related lncRNAs, (**B**) risk model based on the succinylation-related lncRNAs.

PCA revealed the difference of the succinylation-related lncRNAs signature compared with the low- and high-risk specimens based on succinylation-related lncRNAs (Fig. 6A) and the risk model (Fig. 6B) based on the succinylation-related lncRNAs, respectively. These results confirmed the classification ability of the risk signature between the low- and high-risk groups.

### Correlation between the risk score and clinicopathological features

**Figure 7:**
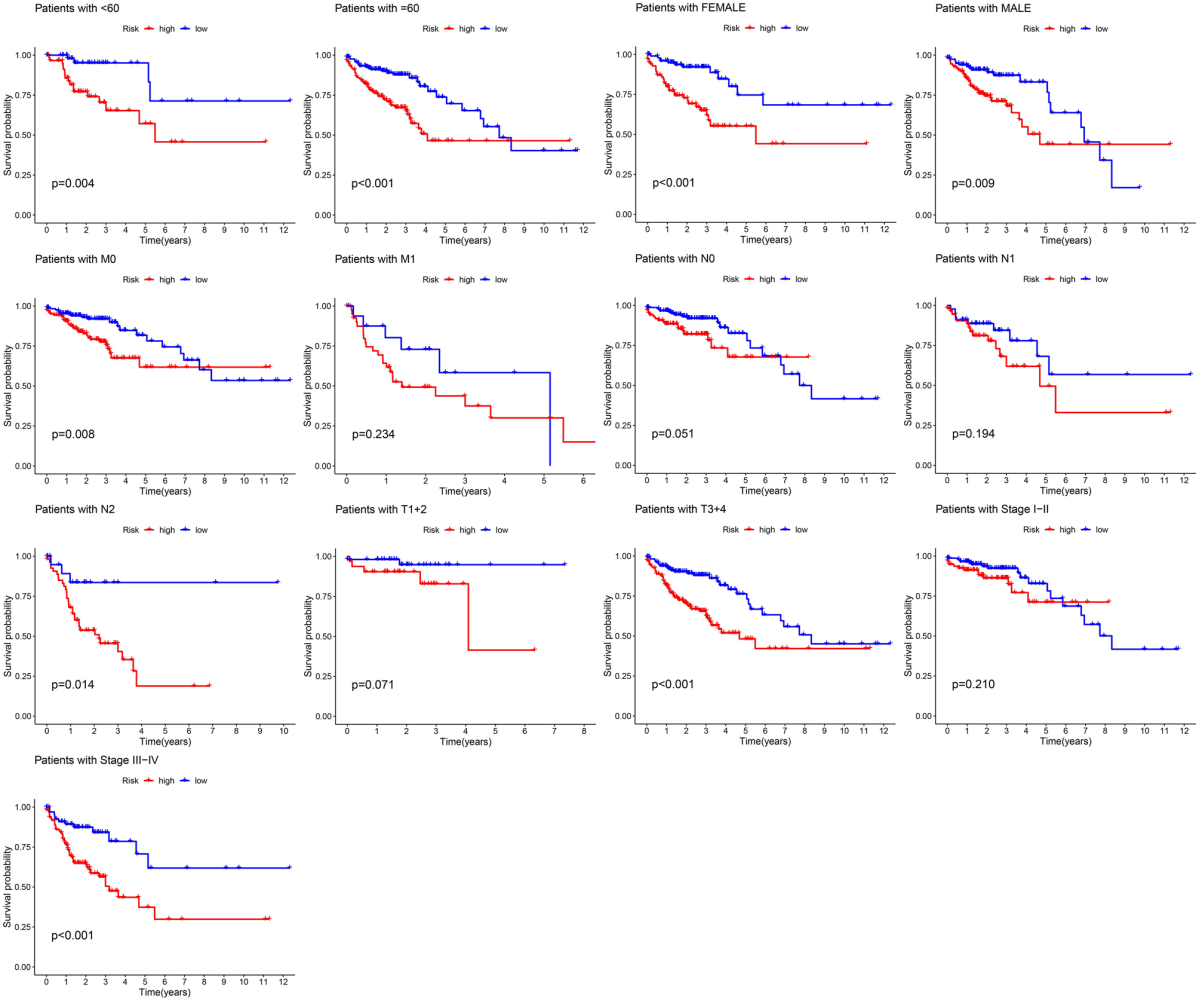
Kaplan–Meier curves by clinical features including age, gender, TNM stage and tumor grade in the high- and low-risk groups.

We analyzed the discrepancies in OS in the high and low-risk groups according to the universal clinicopathologic characteristics and explored the predictive values of succinylation-related lncRNA signature. We classified the subgroups in terms of gender, age and tumor stage (Fig. 7). In the subgroups, the results revealed that succinylation-related lncRNAs signature had a better prognostic value than the M1 stage (p = 0.234, Fig. 7), N0 stage (p = 0.051, Fig. 7), N1 stage (p = 0.194, Fig. 7), Stage I–II (p = 0.21, Fig. 7) and T1 + T2 stage (p = 0.071, Fig. 7. Especially, the OS of patients in the high-risk group was unfavourable (p < 0.05). The study showed that succinylation-related lncRNA signature acted as a novel pivotal indicator to predict the prognosis of CC patients.

### Evaluation of the tumor immune microenvironment and functional enrichment analysis by succinylation-related lncRNA signature

**Figure 8:**
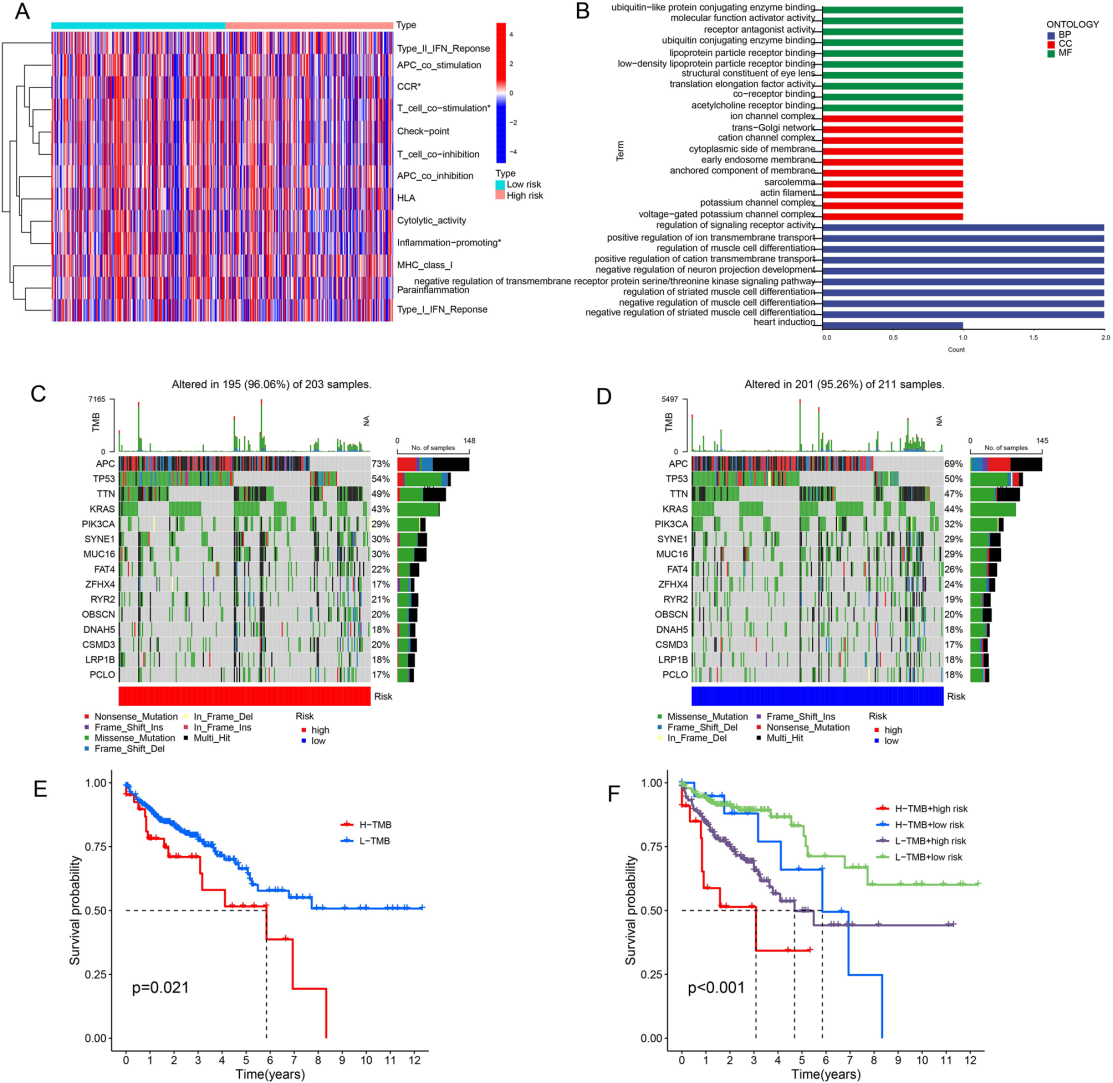
The prognosis tumor immune microenvironment using the model of succinylation-related lncRNAs. (**A**) The standards of the immunity index in patients of CC. (**B**) GO enrichment analysis. (**C** and **D**)Waterfall plot showing the genes mutation information in the high-risk group (**C**) and low-risk group (**D**). (**E** and **F**) Kaplan–Meier analysis for patients based on low- and high- TMB (**E**) and low- and high- TMB with low- and high- risk score (**F**).

To explore the association with succinylation-related lncRNA signature and immune cell infiltration, the heatmap displayed the expression of immune indicators such as CCR, T cells co-stimulation and promotion of inflammation in the high- and low- risk groups (Fig. 8A). To identify the potential biological processes related to the succinylation-related lncRNAs, we used Gene Ontology (GO) enrichment analysis (Fig. 8B). Based on the effect of predicted variance, we then explored the mutation data using the R package maftools. The top 15 genes with the frequency of highest alteration between the high- and low-risk groups were displayed in Fig. 8C and, D, and we found the high-risk group showed a higher mutation frequency than the low-risk group.

Study showed that higher TMB in patients was related to improved response when receiving immune checkpoint blocking therapy^20^. TMB can be seen as a predictive biomarker for cancer immunotherapy. Then, we assessed TMB scores and detected the prognosis correlation with TMB, here, high TMB scores were correlated with a worse survival (Fig. 8E). Whether the succinylation-related lncRNAs model could better predict the OS outcome than TMB scores was analyzed. The high TMB score and low TMB score with CC patients in the high-risk groups (H-TMB of high risk and L-TMB of high risk) displayed a poorer OS than patients with high- and low- TMB score in the low-risk groups (Fig. 8F).

**Figure 9:**
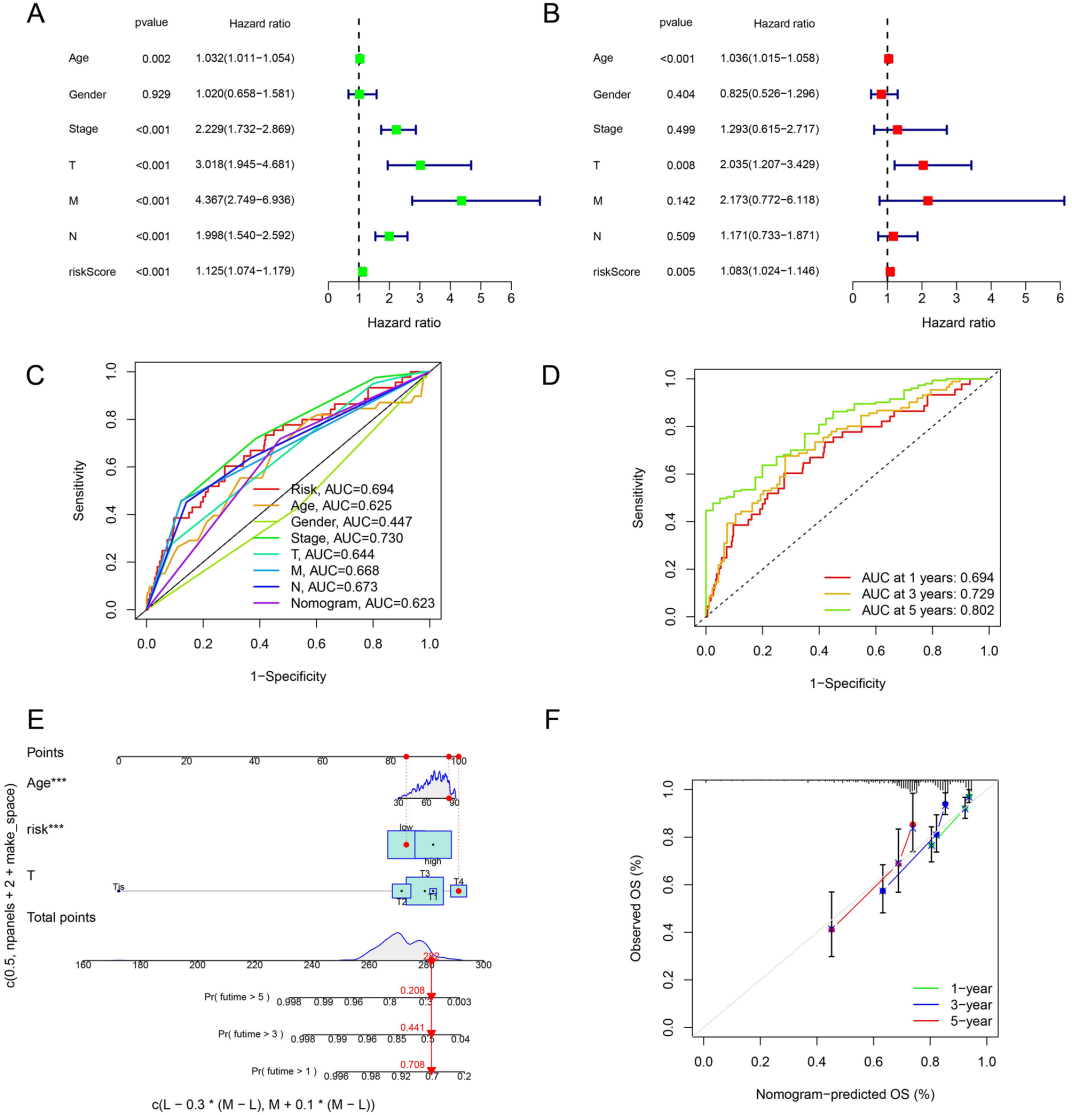
Establishing the association between the succinylation-related lncRNAs and clinical features. (**A**) Univariate and (**B**) multivariate analyses of the clinical features and risk score. (**C**) The risk score concordance indexes with clinical features in CC patients. (**D**) ROC curves in CC patients. (**E**) The nomogram predicts the OS probability. (**F**) The calibration plot predicts the OS probability of the 1-, 3-, and 5-year.

We also analyzed the association with succinylation-related lncRNAs signature and clinical features in patients with CC through the univariate Cox regression analysis in Fig. 9A and multivariate Cox regression analysis in Fig. 9B. As the survival time increased, the risk score index was better, suggesting that the prognosis of patients in CC might be better predicted by the risk model we constructed (Fig. 9C). The ROC curve demonstrated that succinylation-related lncRNA signature to predict the 1-, 3-, and 5-year had an AUC of 0.694, 0.729, and 0.802, respectively (Fig. 9D). The prediction nomogram showed that the overall survival rates could be relatively well predicted across the entire cohort when compared to the ideal model (Fig. 9E and F).

### Screening novel potential compounds targeting succinylation-related lncRNA models

**Figure 10:**
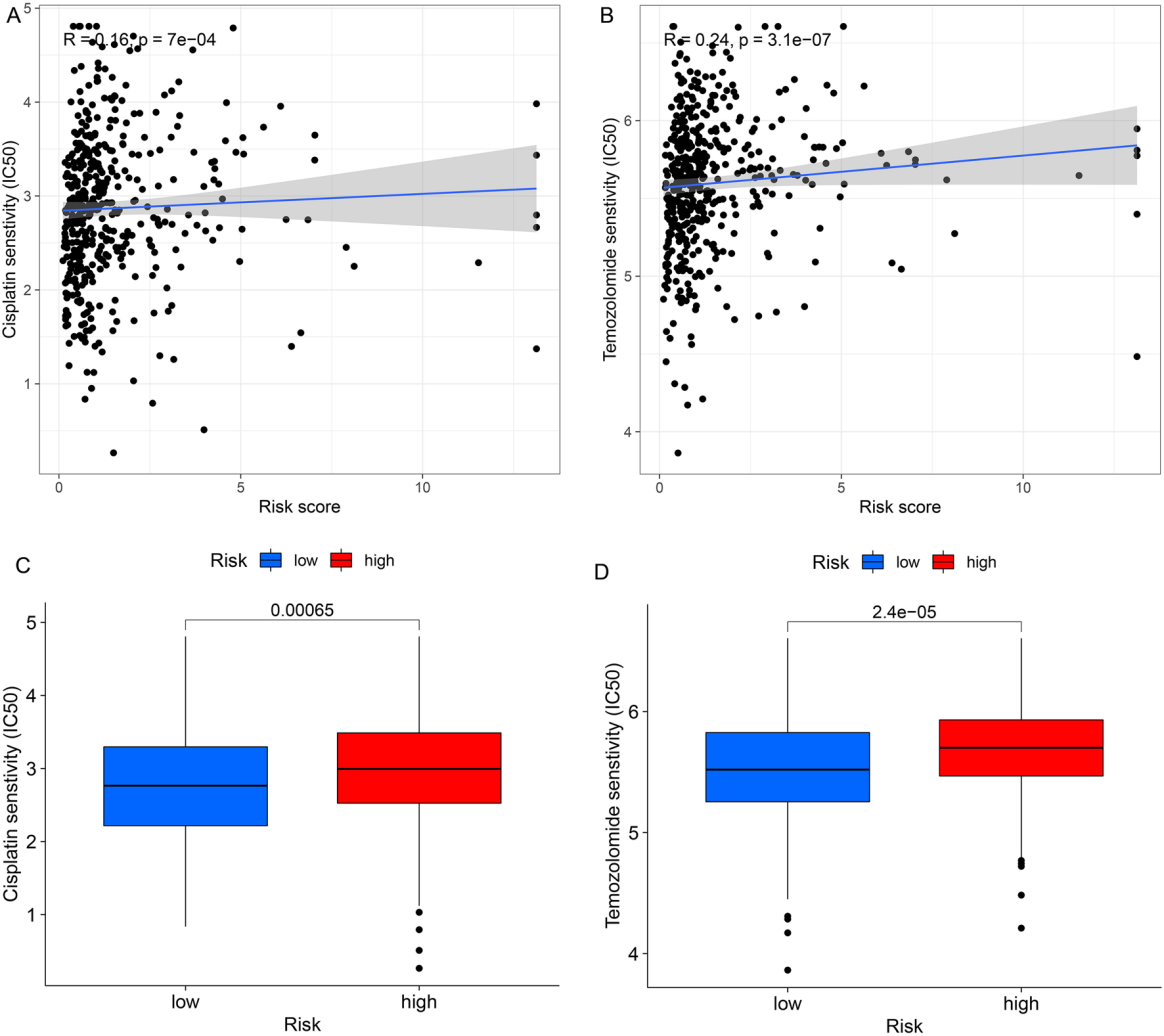
Screening potential drugs in succinylation-related lncRNAs models.

According to the above analysis, to identify potential drugs targeting via succinylation-related lncRNA model for CC patients, we evaluated treatments values according to the half-maximal inhibitory concentration (IC50) by the GDSC database. the Cisplatin and Temozolomide showed positive correlation with the risk score (Fig. 10A, B). It was found that the Cisplatin and Temozolomide compounds showed significantly difference between the two groups (Fig. 10C, D). Besides, . This proved that the current succinylation-related lncRNA was more effective among high-risk patients.

### The difference analysis of microsatellite instability (MSI), TMB and RNA stem score (RNAss) between high group and low group

**Figure 11:**
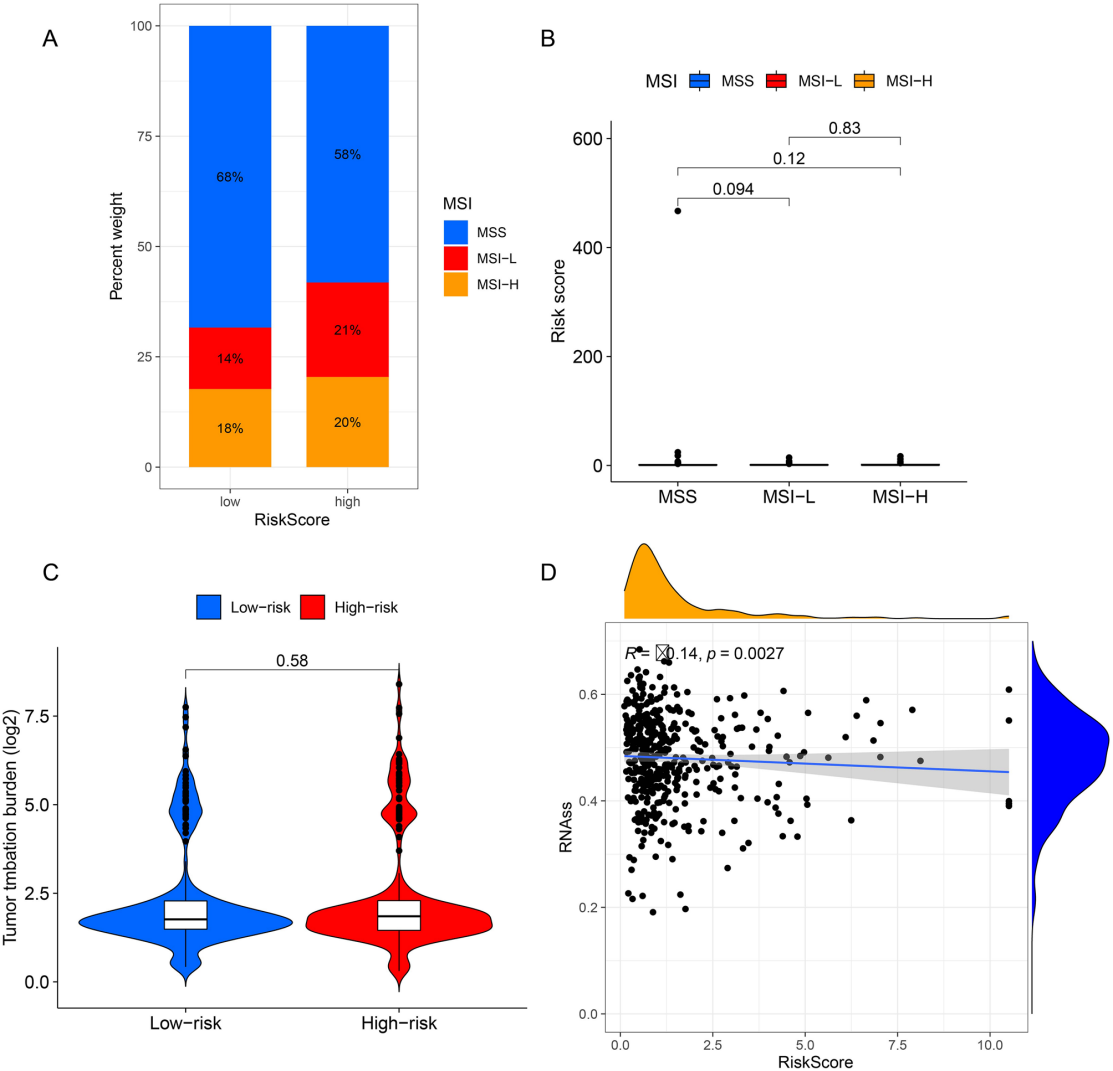
The difference analysis between high group and low group. (**A**) The distribution of MSS and MSI in high- and low- group. (**B**) The difference of RoskScore in MSS, MSI-L and MSI-H group. (**C**) The difference of TMB in high group and low group. (**D**) The correlation analysis between RNAss and RiskSccore.

Immune checkpoint inhibition therapy has shown effective antitumor activity in patients with MSI metastatic cancer and MSS cancers have long been thought to be resistant to immunotherapy. We found that high group had a higher proportion of MSI (Fig. 11A), and but there was no significance in RiskScore among MSS, MSI-L and MSI-H (Fig. 11B). Moreover, the TMB had no difference between high and low group (Fig. 11C). RNAss was correlated with RiskScore (Fig. 11D).

## Discussion

CC as a common clinical gastrointestinal malignancy has a low 5-year survival rate, though clinical treatment techniques and strategies have been improved. We explored the underlying molecular markers for the diagnosis and treatment of CC focusing on developing a succinylation-related lncRNA signature to predict the survival and clinical features in CC patients.

PTMs is an important way to regulate protein function, and its modification forms are greatly diverse and closely related to many tumors. Among of them, succinylation as a novel modification plays an important role in various tumor aspects. In our study, we determined succinylation-related lncRNAs, including FOXD3-AS1, ANK3-DT, EIF3J-DT, MIR210HG, MAFA-AS1 and AC024581.1. Combined with our results, the lncRNA FOXD3-AS1 was overexpressed in glioma, CC and lung cancer^21–23^. In a series of studies, ferroptosis and autophagy-related lncRNA risk models for CC prognosis contained EIF3J-DT^24,25^. Meanwhile, a study found that MIR210HG were associated with survival and metastasis in CC^26^. LncRNAs ANK3-DT, MAFA-AS1 and AC024581.1 were not reported and may be a vital target in CC patient in the future. In this study, based on the succinylation-related lncRNAs, the current signature had a higher accuracy when predicting 1-, 3- and 5-year prognosis of CC patients. Moreover, based on the Kaplan–Meier curves, our risk model showed an excellent stability and reliability to predict the prognosis of CC at the age, gender and pathological grade. These results suggested that succinylation-related lncRNAs should be further studied.

TMB as the number of somatic coding mutations and some genes are frequently mutated in colon tumors. In our study, we revealed that high-risk TMB patient with CC had a poor prognosis. More importantly, we found that the TMB in the low-risk group was related to a better prognosis in CC patients than those in high-risk group. Mutation rates of APC, TP53 and TTN were higher in the high group than in the low group. A study found that APC mutant found in about 80% of all human colon tumor^27^ and enhanced colitis-associated colon carcinogenesis^28,29^. The tumor suppressor gene p53 plays as an important role in the development of various tumors. In CC patients, the most common oncogenic mutation is P53 mutation, accounting for more than 60%^30–32^. A study delineated that the TTN mutant may be a potential predictor in using immune checkpoint inhibitors in lung cancer patients^33,34^. However, the TTN mutant has not been reported in CC. Taken together, we concluded that this predictive model provided reliable immune-biomarkers for tumor therapy.

Co-stimulatory receptor (CCR) was found to be may contribute to target cell docking under some circumstances^35^. There is considerable evidence that combined provision of both CD28 and 4-1BB co-stimulation can synergistically enhance T-cell immune responses^36,37^. We found that CCR and T cell co-stimulation had difference between high risk group and low risk group, indicating those two group may had varying degrees of immunotherapy response.

Nomograms representing the quantitative relationship between multiple risk factors and prognosis is widely used in clinical oncology^38,39^. In our study, by combining clinical features and succinylation-related lncRNA features, we constructed a prognostic nomogram. According to potential drugs targeting, the succinylation-related lncRNAs were associated with the Cisplatin and Temozolomide. Cisplatin and Temozolomide as potential anti-colon cancer drugs have not been reported to be associated with succinylation related lncRNA. Moreover, the succinylation-related lncRNAs for predicting prognosis correlated with the accuracy and clinical value of our nomogram have not been reported. The results also provided a new method to explore the underlying process and mechanism about succinylation modification of lncRNAs. However, the prediction model lacked external data validation and only used the TCGA COAD database. Therefore, we will continue to collect samples for clinical work to explore the exact mechanism in further research.

## Conclusion

In this study, we identified the succinylation-related lncRNAs (FOXD3-AS1, ANK3-DT, EIF3J-DT, MIR210HG, MAFA-AS1 and AC024581.1) that showed the correlation with clinical features in the CC patients. In conclusion, succinylation- related lncRNA model was of great clinical significance for CC patients and might act as a predictive biomarker in the future.

## Data Availability

The data sets analyzed during the current study are available in the TCGA (https://portal.gdc.cancer.gov/). Research data are shared with reasonable request, contact the corresponding author for researchers.
